# Epithelial to Mesenchymal Transition in Human Mesothelial Cells Exposed to Asbestos Fibers: Role of TGF-β as Mediator of Malignant Mesothelioma Development or Metastasis via EMT Event

**DOI:** 10.3390/ijms20010150

**Published:** 2019-01-03

**Authors:** Stefano Turini, Loredana Bergandi, Elena Gazzano, Mauro Prato, Elisabetta Aldieri

**Affiliations:** Department of Oncology, University of Torino, via Santena 5/bis, 10126 Torino, Italy; stefano.turini@unito.it (S.T.); loredana.bergandi@unito.it (L.B.); elena.gazzano@unito.it (E.G.); mauro.prato@unito.it (M.P.)

**Keywords:** epithelial-mesenchymal transition, asbestos, chrysotile, mesothelium, malignant mesothelioma, TGF-β

## Abstract

Asbestos exposure increases the risk of asbestosis and malignant mesothelioma (MM). Both fibrosis and cancer have been correlated with the Epithelial to Mesenchymal Transition (EMT)—an event involved in fibrotic development and cancer progression. During EMT, epithelial cells acquire a mesenchymal phenotype by modulating some proteins. Different factors can induce EMT, but Transforming Growth Factor β (TGF-β) plays a crucial role in promoting EMT. In this work, we verified if EMT could be associated with MM development. We explored EMT in human mesothelial cells (MeT-5A) exposed to chrysotile asbestos: we demonstrated that asbestos induces EMT in MeT-5A cells by downregulating epithelial markers E-cadherin, β-catenin, and occludin, and contemporarily, by upregulating mesenchymal markers fibronectin, α-SMA, and vimentin, thus promoting EMT. In these cells, this mechanism is mediated by increased TGF-β secretion, which in turn downregulates E-cadherin and increases fibronectin. These events are reverted in the presence of TGF-β antibody, via a Small Mother Against Decapentaplegic (SMAD)-dependent pathway and its downstream effectors, such as Zinc finger protein SNAI1 (SNAIL-1), Twist-related protein (Twist), and Zinc Finger E-Box Binding Homeobox 1 (ZEB-1), which downregulate the *E-cadherin* gene. Since *SNAIL-1*, *Twist*, and *ZEB-1* have been shown to be overexpressed in MM, these genes could be considered possible predictive or diagnostic markers of MM development.

## 1. Introduction

Asbestos is the common name for a group of hydrated fibrous silicates, whose exposure has been held responsible for a large number of lung diseases, such as asbestosis (a form of asbestos-induced fibrosis), lung cancer, and malignant mesothelioma (MM) [1]. No single mechanism fully accounts for all the complex toxic and carcinogenic effects caused by asbestos [2].

Both pulmonary fibrosis and tumors have been associated with the ability of epithelial cells to become mesenchymal cells through a process known as Epithelial to Mesenchymal Transition (EMT). EMT is both a physiological and pathological process: it is related to embryonic development, later organogenesis, as well to wound healing in fibrotic tissues, in tumor development, and metastasis [3,4]. During EMT, cells lose the epithelial phenotype associated with a decrease in protein epithelial markers, such as the adherens junctions, E-cadherin, and β-catenin, and the tight junction protein ZO-1 (Zonula Occludens). By contrast, these cells acquire a mesenchymal phenotype correlated with an increased production of mesenchymal markers such as fibronectin, vimentin and α-SMA (Smooth Muscle Actin) [5,6,7,8]. These biochemical events involve some conformational cellular changes through which cells acquire a fibroblast-like morphology [6,9,10].

The spectrum of changes that occur during EMT depends on several factors such as the microenvironment surrounding the cells and the type of inductor. Among the extracellular signals able to induce EMT, there are some growth factors, such as Transforming Growth Factor β (TGF-β), hepatocyte growth factor (HGF), and cytokines, such as tumor necrosis factor-α (TNF-α) [6,10,11,12]. TGF-β is crucial in EMT events [6] and is able to regulate cell growth and differentiation, as well as cell transformation and carcinogenesis [13,14]. Many studies reported in the literature have correlated the toxic effects of asbestos with increased TGF-β secretion, thus promoting an inflammatory status and driving the development of pulmonary fibrosis [15]. Some authors investigated the effects of asbestos and its role in EMT. Tamminen et al. [16] showed that asbestos can induce EMT in lung epithelioma A549 cells: in their experiments, they exposed cultured human lung epithelial cells to crocidolite asbestos and analyzed alterations in the expression of epithelial and mesenchymal marker proteins and cell morphology. Asbestos was found to induce downregulation of E-cadherin in A549 cells [16], loss of cell–cell contacts, and actin reorganization, and upregulation of α-Smooth Muscle Actin (α-SMA). Qi et al. [17] suggested that continuous exposure to crocidolite and chrysotile asbestos could cause EMT in human mesothelial cells via High Mobility Group Box 1 (HMGB1) and TNF-α signaling [17].

Given the strong association of asbestos exposure with TGF-β activation [18,19], our research group [20] already demonstrated the role of chrysotile asbestos in inducing EMT in human bronchial epithelial cells (BEAS-2B), via TGF-β and its intracellular effectors Protein Kinase B (PKB or Akt), Glycogen synthase kinase 3 beta (GSK-3β), and Zinc finger protein SNAI1 (SNAIL-1). TGF-β is responsible for the activation of a canonical pathway mediated by the intracellular effectors Small Mother Against Decapentaplegic (SMAD) proteins [21], which in turn induces downstream effectors responsible for EMT markers modulation.

Various studies have explored the role of EMT in MM: Casarsa et al. [22] showed the importance of EMT markers in MM prognosis, and others [22,23] evaluated the prognostic value of EMT markers in MM. Kim et al. [24] proposed HIF-1α as a mediator of MM transformation via EMT event.

In the present study we investigated the role of TGF-β in EMT induction of chrysotile in human mesothelial cells (MeT-5A) in order to identify a possible molecular mechanism associated with malignant mesothelioma development after asbestos exposure.

## 2. Results

### 2.1. Asbestos Fibers Induce Fibroblastoid Morphological Changes in MeT-5A Cells

**Figure 1:**
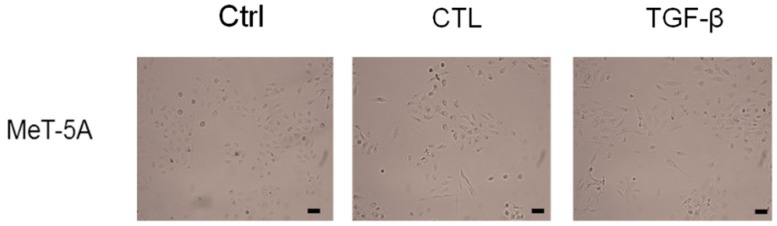
Effects of chrysotile asbestos or TGF-β on cell morphology. MeT-5A cells were cultured in the absence (Ctrl) or presence of 5 µg/cm^2^ chrysotile asbestos (CTL) or 10 ng/mL TGF-β for 72 h of incubation. MeT-5A cells changed their morphology from the typical shape of untreated cells to a spindle-shaped fibroblast-like morphology typical of an EMT event. 10×; scale bar = 50 μm.

MeT-5A cells were incubated as described in Materials and Methods. After incubation with chrysotile asbestos fibers (CTL) or TGF-β, cells acquired a characteristic fibroblastoid morphology typical of EMT events (Figure 1) and appeared more elongated and thinner compared to untreated cells (Ctrl).

### 2.2. Chrysotile Asbestos Downregulates Epithelial Markers and Upregulates Mesenchymal Markers in MeT-5A Cells

**Figure 2:**
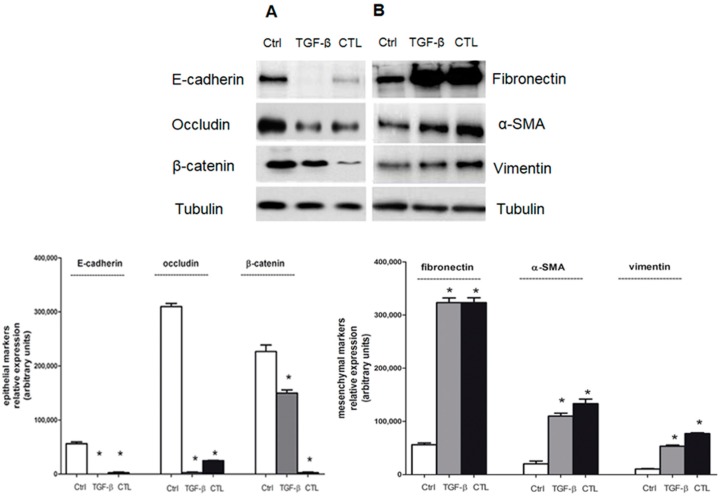
Effects of chrysotile asbestos on EMT marker protein levels in MeT-5A cells. MeT-5A cells were cultured for 72 h without (Ctrl) or with 5 μg/cm^2^ chrysotile (CTL) or 10 ng/mL TGF-β. (**A**) Expression of epithelial (E-cadherin, occludin, and β-catenin) and (**B**) mesenchymal (fibronectin, α-SMA, and vimentin) markers checked by Western blotting. Tubulin was used as a loading control. The image is representative of three independent experiments that produced similar results. Densitometry data are presented as the percent decrease or increase in the protein levels versus the respective control. Significance versus the respective control: * *p* < 0.001.

MeT-5A cells, exposed to CTL asbestos fibers or TGF-β, showed a significant decrease in the level of E-cadherin—the main epithelial marker in EMT—unlike untreated cells (Figure 2A). MeT-5A cells exposed to CTL or TGF-β also exhibited a downregulation of β-catenin and occludin (Figure 2A), thus confirming the start of EMT induction. In parallel, MeT-5A cells exposed to CTL or TGF-β showed a significant upregulation of mesenchymal markers fibronectin, vimentin, and α-SMA, unlike untreated cells (Figure 2B), thus confirming the EMT occurrence.

**Figure 3:**
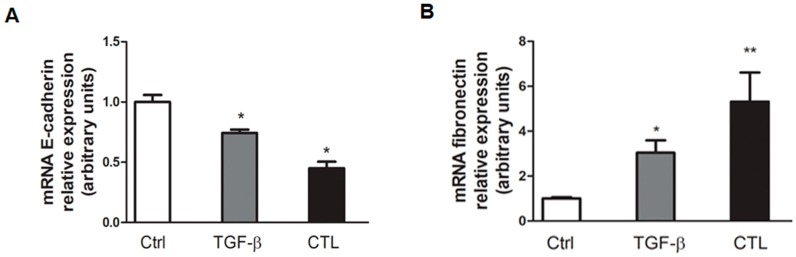
Relative gene expression of *E-cadherin* and *fibronectin* after asbestos exposure. *E-cadherin* and *fibronectin* mRNA content was evaluated by quantitative real-time polymerase chain reaction (qRT-PCR). Data are expressed in units of relative mRNA expression compared with control cells (*n* = 3). Significance versus the respective control: * *p* < 0.005; ** *p* < 0.001.

In order to highlight the changes in gene expression, the same pattern in EMT marker modulation was observed in mRNA transcription evaluation. There was a greater decrease in *E-cadherin* mRNA expression and a simultaneous significant increase in mRNA *fibronectin* content after CTL or TGF-β incubation (Figure 3), thus confirming our previous Western blotting data.

### 2.3. Chrysotile Increases MMP-2 Secretion While EMT Event Was Induced

**Figure 4:**
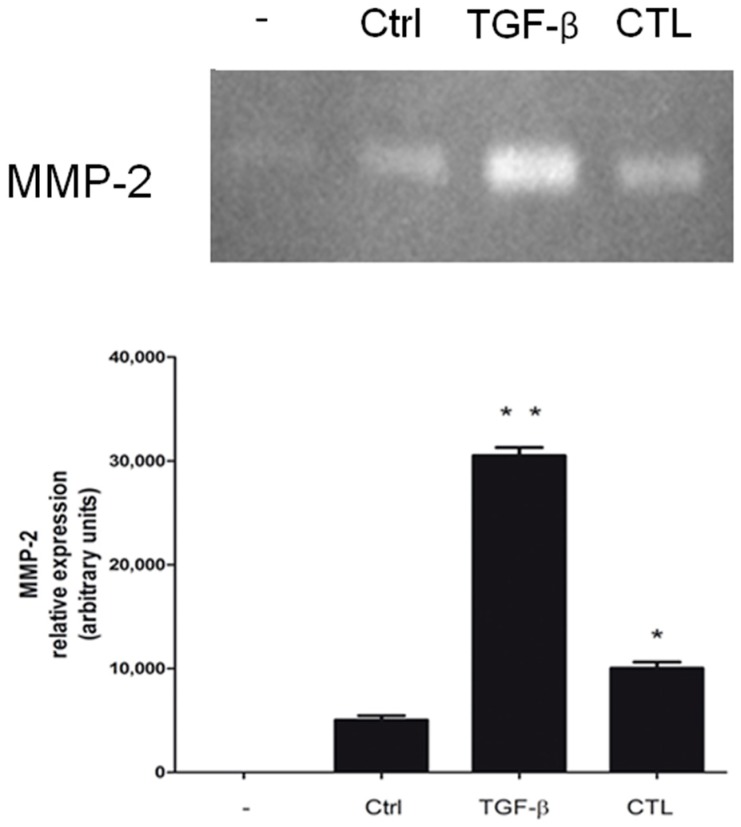
Effect of chrysotile asbestos on MMP-2 secretion and activation. MeT-5A cells were cultured for 72 h without (Ctrl) or with 5 μg/cm^2^ chrysotile (CTL) or 10 ng/mL TGF-β. At the end of the incubation, the levels of MMP-2 were measured in the cell supernatants after normalization. Measurements were performed in triplicate and data are presented as means ± SEM (*n* = 3). Significance versus the respective control: * *p* < 0.05; ** *p* < 0.01.

Since Matrix Metalloproteases (MMP) play a key role in the remodeling of the extracellular matrix and MMP-2 is a well-known marker of EMT, we investigated its secretion and activity. We observed that MeT-5A cells exposed to CTL or TGF-β excreted more MMP-2 compared with untreated cells (Figure 4).

### 2.4. Exposure to Chrysotile Asbestos Increases TGF-β Secretion in MeT-5A Cells and Co-Incubation with Anti-TGF-β Antibody Restores Basal Expression Level of EMT Markers

Chrysotile asbestos exposure has already been associated with an increased secretion of the TGF-β [15] and our research group demonstrated this event in pulmonary BEAS-2B cells exposed to chrysotile [20].

**Figure 5:**
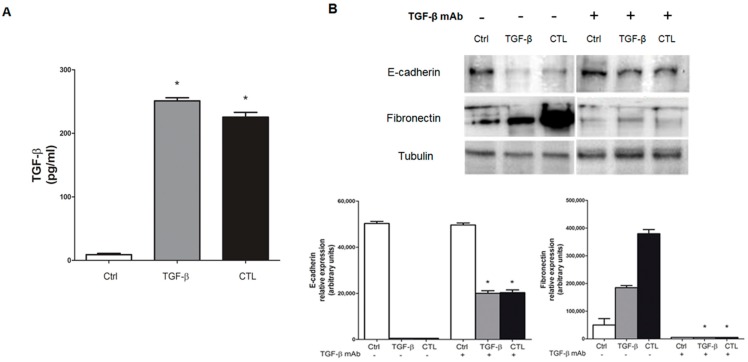
TGF-β secretion and neutralizing TGF-β antibody effect in MeT-5A cells exposed to chrysotile. MeT-5A cells were cultured for 72 h without (Ctrl) or with 5 μg/cm^2^ chrysotile (CTL) or 10 ng/mL TGF-β for 72 h. (**A**) After incubation, the supernatants were collected and TGF-β levels were detected using an ELISA kit. Data are shown as the mean ± SEM (*n* = 3). TGF-β levels are reported as picograms per milligram of intracellular protein. Significance versus the respective control: * *p* < 0.001. (**B**) MeT-5A cells were incubated without (Ctrl) or with 5 μg/cm^2^ chrysotile (CTL) or 10 ng/mL TGF-β, and with CTL or TGF-β and 5 ng/mL of neutralizing anti-TGF-β antibody for 72 h. The expression of epithelial (E-cadherin) and mesenchymal (fibronectin) markers was determined by Western blotting. Tubulin was used as a loading control. The image is representative of three independent experiments. Densitometry data are presented as the percent decrease or increase versus control cells. Significance versus the respective control: * *p* < 0.001.

TGF-β levels were measured in MeT-5A cells exposed to CTL asbestos, and our results showed a significant increase in TGF-β secretion (Figure 5A). Then, cells were co-incubated with the neutralizing anti-TGFβ antibody to confirm TGF-β is the mediator of the reported EMT markers changes. As shown in Figure 5B, E-cadherin was significantly decreased and fibronectin increased in cells treated with chrysotile asbestos (CTL), whereas the co-incubation of cells with TGF-β blocking antibody restored these protein expression levels (Figure 5B).

### 2.5. Exposure to Chrysotile Induces E-Cadherin Downregulation Through SMAD Pathway via Increased Secretion of TGF-β

**Figure 6:**
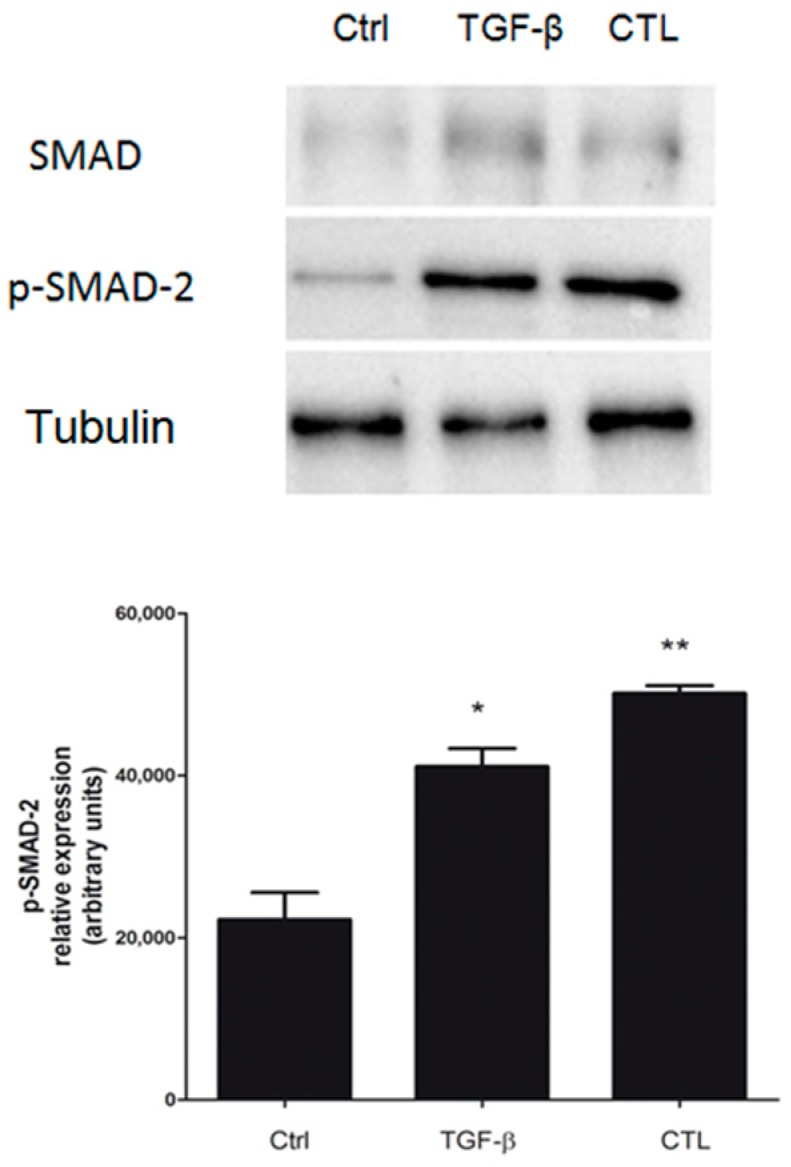
Evaluation of the role of the TGF-β mediated SMAD-dependent pathway in MeT-5A cells exposed to asbestos. MeT-5A cells were cultured for 72 h without (Ctrl) or with 5 μg/cm^2^ chrysotile (CTL) or 10 ng/mL TGF-β for 72 h. The expressions of SMAD-2 and active p-SMAD-2 factors were determined by Western blotting. Tubulin was used as a loading control. The image is representative of three independent experiments. Densitometry data are presented as the percent increase of p-SMAD versus control cells. Significance versus the respective control: * *p* < 0.05; * *p* < 0.01.

As shown above, chrysotile asbestos drove EMT by increasing the secretion of TGF-β from MeT-5A cells. Once TGF-β binds its receptor, the recruitment of phosphorylated SMAD-2/SMAD-3 proteins occurs [21]: the phosphorylated SMAD-2 protein binds SMAD-4 to form a SMAD heterocomplex that mediates signal transduction [21]. In the present work, the involvement of the TGF-β-mediated SMAD-dependent canonical pathway in MeT-5A cells exposed to asbestos was confirmed. Our results demonstrated that the CTL, such as TGF-β alone, increased basal SMAD-2 phosphorylation and, consequently, its activation (Figure 6).

### 2.6. EMT Induced by Chrysotile is Mediated by GSK-3β/SNAIL-1 Activation

**Figure 7:**
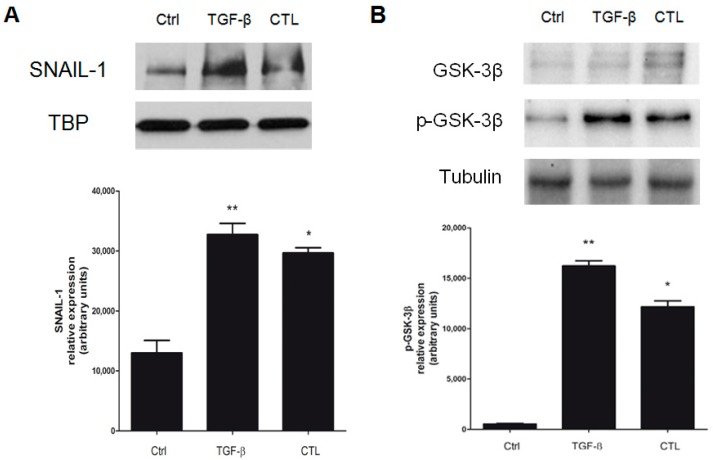
Evaluation of the role of the GSK-3β/SNAIL-1 pathway in E-cadherin modulation in MeT-5A cells. The images are representative of three independent experiments that produced similar results. MeT-5A cells were incubated in the absence (Ctrl) or presence of 5 μg/cm^2^ chrysotile (CTL) or 10 ng/mL TGF-β for 72 h. (**A**) The accumulation of SNAIL-1 in the nuclei of MeT-5A cells were examined by Western blotting. Experiments were performed in triplicate. TATA-binding protein (TBP) was used as loading control for the nucleus. Densitometry data concerning SNAIL-1 accumulation in the nuclei are presented as the percent increase in the protein levels versus control. Significance versus control: * *p* < 0.005; ** *p* < 0.001. (**B**) The expression of phosphorylated (p-GSK-3β) and basal GSK-3β are shown. Tubulin was used as loading control. Experiments were performed in triplicate, and densitometry data are presented for p-GSK-3β as the percent increase in the protein levels versus the respective control. Significance versus the respective control: * *p* < 0.001; ** *p* < 0.0001.

The SMAD-2 phosphorylation event induces some transcription factors such as SNAIL-1, which in turn promotes E-cadherin downregulation and, consequently, the start of EMT [25]. In MeT-5A cells treated with CTL or TGF-β, nuclear SNAIL-1 resulted translocated into the nucleus, and not in untreated cells (Figure 7A). In particular, in our previous work [20], we demonstrated that SNAIL-1 factor, in bronchial BEAS-2B cells exposed to chrysotile, is crucial in downregulating E-cadherin when it is accumulated into the nucleus, and this mechanism is mediated by a GSK-3β-dependent mechanism [26]. GSK-3β phosphorylation, and its consequent inactivation, allows SNAIL-1 to accumulate in the nucleus, where it downregulates the *E-cadherin* gene. Our results confirm phosphorylation of GSK-3β drives SNAIL-1 accumulation in the nucleus (Figure 7B), thus inhibiting E-cadherin expression.

### 2.7. Twist and ZEB-1 Factors Are Involved in EMT via TGF-β in MeT-5A Cells Exposed to Asbestos Fibers

**Figure 8:**
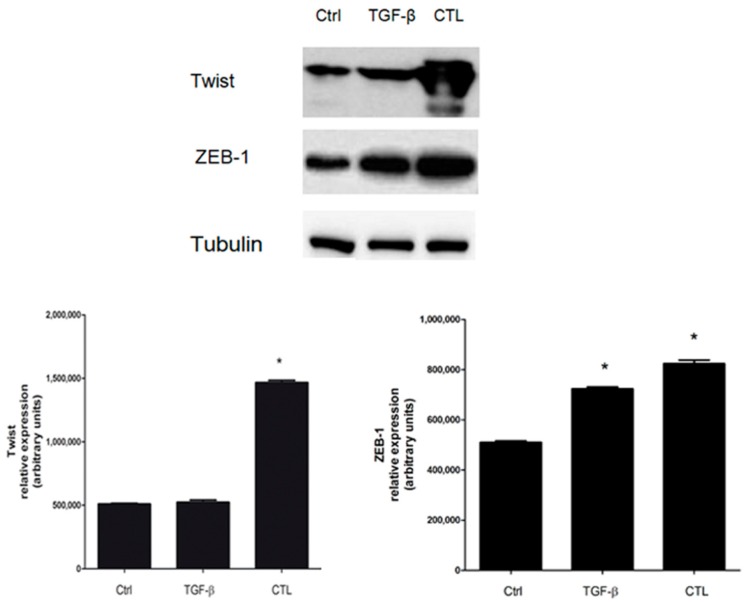
Evaluation of the role of Twist and ZEB-1 factors in *E-cadherin* gene downregulation. MeT-5A cells were incubated in the absence (Ctrl) or presence of 5 μg/cm^2^ chrysotile (CTL) or 10 ng/mL TGF-β for 72 h. The expression of Twist and ZEB-1 was determined by Western blotting. Experiments were performed in triplicate. Tubulin was used as loading control. Densitometry data are presented as the percent decrease or increase versus control cells. Significance versus the respective control: * *p* < 0.005.

Besides SNAIL-1, TGF-β, via a SMAD-dependent pathway, mediates the activation of Twist and ZEB-1 factors, which in turn can downregulate E-cadherin expression [27]. In our experiments in MeT-5A cells incubated with CTL or TGF-β, we observed that both Twist and ZEB-1 were overexpressed in untreated cells. We simultaneously observed a downregulation of E-cadherin, thus confirming the EMT event (Figure 8).

## 3. Discussion

The actual problem with asbestos related-pathologies, such as asbestosis and malignant mesothelioma (MM), is identifying predictive markers of exposure or pharmacological targets useful for developing a fast approach toward diagnosing these severe pathologies, in particular concerning MM. Fibrosis and cancer involved in their pathological mechanism a common multistep event called Epithelial-Mesenchymal Transition (EMT), which plays a fundamental role in development/invasiveness mainly in pulmonary tumors [9].

Few papers in the literature focused on the ability of asbestos fibers to induce EMT: Tamminen et al. [16] carried out a study on A549 epithelioma cells using crocidolite asbestos, and the authors demonstrated that crocidolite is able to induce EMT through a mechanism involving the Mitogen-activated protein kinase/Extracellular signal-regulated kinase (MAPK/Erk) signaling pathway. As previously reported by our research group, chrysotile asbestos induces EMT in human bronchial BEAS-2B cells [20], and this event could be associated with asbestosis development. Accordingly, we addressed our goal of verifying if this mechanism could be also correlated with MM development and/or invasiveness, via an EMT event, in mesothelial cells exposed to asbestos fibers. In our cellular model, chrysotile asbestos fires incubated in Met-5A cells were able to induce a phenotypic transition, morphologically associated with an EMT event. EMT is known to be a process that can be triggered by many different molecular mechanisms and in many cellular models. However, despite this important evidence, we have not yet elucidated a clear mechanism connecting asbestos effects and EMT induction. Our evidence confirms data already present in the literature: Qi et al. [17] stated the morphological and molecular alterations induced by crocidolite and chrysotile asbestos are suggestive of EMT in mesothelial cells.

We confirmed the morphological data by evaluating EMT markers in Met-5A cells exposed to chrysotile asbestos compared with untreated cells: asbestos fibers downregulated epithelial markers such as E-cadherin, occludin, and β-catenin, while simultaneously upregulated mesenchymal markers such as fibronectin, α-SMA, and vimentin, thus confirming the EMT start in MeT-5A cells.

TGF-β is one of the main EMT inducers and it has already been reported to mediate asbestos-induced fibrosis and inflammation [19,28]. In our cellular model, we evaluated TGF-β secretion using the ELISA method to demonstrate the involvement of TGF-β in mediating EMT events observed in Met-5A cells. We observed an increased and significant production of TGF-β after asbestos incubation, and this crucial involvement was confirmed by a co-incubation of mesothelial cells with TGF-β-neutralizing antibody. The blocking antibody restored the expression levels of epithelial E-cadherin and mesenchymal fibronectin markers, suggesting the crucial role of TGF-β. Kim et al. [3] analyzed reactive oxygen species (ROS)-induced EMT in human malignant mesothelioma cells, suggesting that oxidative stress induced by H_2_O_2_ may play a critical role in mesothelioma carcinogenesis via TGF-β, HIF-1α, and some stemness genes [3].

To expand our knowledge about the molecular mechanisms proposed, we referred to our previous work where we reported TGF-β to be responsible for the up-regulation of the SMAD-dependent pathway to drive downregulation of the main EMT marker, E-cadherin [20]. Moreover, the SMAD-dependent TGF-β mediated pathway is known to drive the EMT event [29].

Our results show an increased expression of SMAD-2 in Met-5A cells exposed to asbestos fibers or TGF-β, confirming the involvement of a SMAD-dependent pathway in EMT. SMAD activation can promote a wide spectrum of downstream effector, directly related to the modulation of EMT markers [29]. Among these factors, SNAIL-1, Twist, and ZEB-1 all play a pivotal role in downregulating E-cadherin expression [21]. We observed that all these factors were strongly activated or overexpressed after asbestos exposure in MeT-5A cells.

Among these factors, transcription factor SNAIL-1 was particularly associated in our previous work with EMT, and SNAIL-1 was in turn implicated in *E-cadherin* gene downregulation, as demonstrated [20]. To exert this function, SNAIL-1 persistence into the nucleus is required [26]. We observed the accumulation of SNAIL-1 into the nucleus of MeT-5A cells incubated with asbestos fibers as a consequence of the phosphorylation and inactivation of GSK-3β, thus allowing SNAIL-1 to translocate into the nucleus and to repress E-cadherin transcription [20,30]. We detected an increased phosphorylation of GSK-3β accompanied by the simultaneous accumulation of SNAIL-1 in the nucleus. Therefore, with the present data, we demonstrated for the first time that chrysotile asbestos induces EMT in MeT-5A cells with a molecular mechanism involving TGF-β and its intracellular effectors, GSK-3β and SNAIL-1. However, the non-total recovery of the investigated epithelial and mesenchymal markers can be explained considering TGF-β as just one of the possible mediators of asbestos-induced EMT events in these cells.

SNAIL-1, ZEB-1, and Twist factors have been shown to play a role in malignant mesothelioma (MM), as demonstrated by Merikallio et al. [31]. The authors demonstrated that all these factors were overexpressed in MM, highlighting their significance in the metastatic process. Our data demonstrated an overexpression of SNAIL-1, Twist, and ZEB-1 after chrysotile exposure in Met-5A cells, with a parallel and effective downregulation of E-cadherin. Thus, it is conceivable to hypothesize that this mechanism is involved in MM development and/or metastasis via an EMT event.

TGF-β signaling has been extensively associated with lung cancer development [32], particularly related to an increased inflammatory microenvironment formation and a consequent deregulation of some oncogenes, such as *P53* [33]. Burmeister et al. [34] demonstrated a P53 overexpression in Met-5A cells exposed to asbestos fibers, as a condition related to the nature of this cell line as immortalized with SV40. Thus, TGF-β may conceivable also have a role in a cross-talk with P53 in our cellular model and in a synergistic effect to promote MM development, so increasing the complexity of TGF-β-mediated events.

Finally, the increased deposition of fibronectin and the extrusion of MMP-2 in cells exposed to chrysotile asbestos suggest important changes in the surrounding microenvironment, which make the extracellular matrix more suitable to be degraded and invaded [35]. However, the mechanism proposed can be considered as just one of the possible molecular mechanisms supporting the morphological transformations typical of EMT involved in MM development.

## 4. Materials and Methods

### 4.1. Asbestos Samples

Union International Contre le Cancer (UICC) chrysotile was sonicated (100 W, 30 s, Labsonic Sonicator; Sartorius Stedim Biotech S.A., Goettingen, Germany) before incubation with cell cultures to dissociate fiber bundles and to improve their suspension in the culture medium.

### 4.2. Cell Cultures

Human mesothelial cells (MeT-5A) were obtained from American Type Culture Collection (ATCC^®^ CRL-9444^TM^, ATCC, Manassas, VA, USA). They were cultured in Medium 199 (M199) (Gibco, Paisley, UK) supplemented with 10% fetal bovine serum (FBS) and 1% penicillin/streptomycin.

Cell cultures were maintained in a humidified incubator at 37 °C in a 5% CO_2_ atmosphere.

### 4.3. Experimental Conditions

Dose- and time-dependence experiments were performed to determine the appropriate concentration and time, respectively, for incubating MeT-5A cells with chrysotile. As a consequence of these preliminary results, 4.5 × 10^5^ cells in 100-mm-diameter Petri dishes were incubated for 72 h in the absence or presence of 5 µg/cm^2^ of chrysotile asbestos suspension or 10 ng/μL TGF-β solution in M199 medium. The protein content in the cells was detected using a bicinchoninic acid assay (BCA) kit (Sigma Chemical Co., Saint Louis, MO, USA). The plasticware for cell culture was provided by Falcon (Corning Incorporated, Corning, NY, USA). Ultrapure water (Millipore, Burlington, MA, USA) was used for all experiments. A solution of recombinant TGF-β was obtained by re-suspending the lyophilized form of Human Recombinant TGF-β (PeproTech, Rocky Hill, CT, USA) in a 10 mM citric acid solution, with a pH of 3.0 in order to increase the storage time. The solution was then diluted in a 0.1% BSA-based buffer.

### 4.4. Cell Morphology

At the end of the incubation period, cells were observed using a light microscope, and images were obtained with the Leica Application Suite program (Leica Microsystems, Wetzlar, Germany).

### 4.5. Western Blot Analysis

Cytosolic and nuclear extracts were obtained using an Active Motif nuclear extraction kit (Active Motif, La Hulpe, Belgium) according to the manufacturer’s instructions. Cytosolic and nuclear extracts were separated by sodium dodecyl sulfate-polyacrylamide gel electrophoresis (SDS-PAGE), transferred to polyvinylidene difluoride (PVDF) membrane sheets (Immobilon-P, Millipore) and probed with the required antibody diluted in 0.1% PBS-Tween with 5% nonfat dry milk. After 1 h of incubation, the membranes were washed with 0.1% PBS-Tween and then incubated for 1 h with peroxidase-conjugated sheep anti-mouse or sheep anti-rabbit IgG antibody (Amersham International, Little Chalfont, UK) diluted 1:3000 in 0.1% PBS-Tween with 5% nonfat dry milk. The membranes were washed again with 0.1% PBS-Tween, and proteins were detected by enhanced chemiluminescence (Perkin Elmer, Waltham, MA, USA). Antibodies against E-cadherin, fibronectin, β-catenin, occludin, tubulin, TATA-binding protein (TBP), SMAD, SNAIL-1, Twist, and ZEB-1 were all provided by Santa Cruz Biotechnology, Inc. (Santa Cruz, CA, USA). The anti-vimentin antibody was provided by Sigma Chemical Co. The anti-α-SMA antibody was from GeneTex (Irvine, CA, USA). The anti-SMAD-2 and p-SMAD-2 antibodies were from Abcam (Cambridge, UK). The neutralizing anti-TGF-β antibody was purchased from Abcam and was used at a concentration of 5 μg/mL. Tubulin and TBP were used as loading controls for the cytosol and the nucleus, respectively.

### 4.6. Quantitative Real-Time Polymerase Chain Reaction (qRT-PCR)

Total RNA was obtained by the guanidinium thiocyanate–phenol–chloroform method (Chomczynski and Sacchi 1987), using RiboZol RNA Extraction Reagents (Amresco) according to the manufacturer’s instructions. Total RNA (0.2 μg) was reverse-transcribed into cDNA using an iScript cDNA Synthesis Kit (Bio-Rad Laboratories AG, Cressier FR, Switzerland) according to the manufacturer’s instructions. qRT-PCR was performed using IQ™ SYBR Green Supermix (Bio-Rad) according to the manufacturer’s instructions. PCR amplification was performed as follows: 1 cycle of denaturation at 94 °C for 3 min, 45 cycles of denaturation at 94 °C for 30 s, annealing for 30 s, and synthesis at 72 °C for 30 s.

The relative expression of each target gene was determined by comparing each PCR gene product with the S14 ribosomal subunit product using the Gene Expression Macro (http://www3.bio-rad.com/LifeScience/jobs/2004/04-0684/genex.xls; Bio-Rad).

### 4.7. Quantification of TGF-β Secretion by ELISA

After incubating MeT-5A cells in the experimental conditions described above, the extracellular medium was collected and centrifuged at 4 °C at 13,000× *g* for 30 min. To determine the concentration of TGF-β in the supernatant, ELISA was performed according to the manufacturer’s instructions (Invitrogen Corporation, Carlsbad, CA, USA). Absorbance was measured at 450 nm with a Synergy HT microplate reader (BioTek, Winooski, VT, USA). The amount of cytokine was determined using a standard curve and was corrected for the content of cell protein. The results were expressed as pg/mg of intracellular protein.

### 4.8. Gelatin Zymography

Because FBS contains matrix metalloproteinases (MMPs), cells were only cultured in 1% serum medium. Afterward, the supernatant was collected, supplemented with Laemmli sample buffer, and subjected to 10% SDS-PAGE with 1 mg/mL gelatin under non-denaturing and non-reducing conditions as previously described [36].

### 4.9. Statistical Analysis

Where appropriate, data in figures are reported as the mean±SEM. The results were analyzed by one-way analysis of variance (ANOVA) and Tukey’s test (SPSS 11.0 for Windows; SPSS Inc., Chicago, IL, USA); *p* < 0.05 was considered significant.

## 5. Conclusions

Overall, it is conceivable to consider this new molecular approach in an attempt to not only identify new predictive markers but also to provide a new treatment strategy for *SNAIL*-, *Twist*-, and *ZEB*-overexpressing MM.

## References

[1] Kamp D.W. (2009). Asbestos-induced lung diseases: An update. Transl. Res..

[2] Kamp D.W., Weitzman S.A. (1999). The molecular basis of asbestos induced lung injury. Thorax.

[3] Kalluri R., Neilson E.G. (2003). Epithelial-mesenchymal transition and its implications for fibrosis. J. Clin. Investig..

[4] Lamouille S., Xu J., Derynck R. (2014). Molecular mechanisms of epithelial-mesenchymal transition. Nat. Rev. Mol. Cell Biol..

[5] Dongre A., Weinberg R.A. (2018). New insights into the mechanisms of epithelial-mesenchymal transition and implications for cancer. Nat. Rev. Mol. Cell Biol..

[6] Cannito S., Novo E., Di Bonzo L.V., Busletta C., Colombatto S., Parola M. (2010). Epithelial-mesenchymal transition: From molecular mechanisms, redox regulation to implications in human health and disease. Antioxid. Redox Signal.

[7] Boyer B., Vallés A.M., Edme N. (2000). Induction and regulation of epithelial-mesenchymal transitions. Biochem. Pharmacol..

[8] Peinado H., Portillo F., Cano A. (2004). Transcriptional regulation of cadherins during development and carcinogenesis. Int. J. Dev. Biol..

[9] Kalluri R., Weinberg R.A. (2009). The basics of epithelial-mesenchymal transition. J. Clin. Investig..

[10] Moustakas A., Heldin C.H. (2012). Induction of epithelial-mesenchymal transition by transforming growth factor β. Semin. Cancer Biol..

[11] Chen J., Chen G., Yan Z., Guo Y., Yu M., Feng L., Jiang Z., Guo W., Tian W. (2014). TGF-β1 and FGF2 stimulate the epithelial-mesenchymal transition of HERS cells through a MEK-dependent mechanism. J. Cell Physiol..

[12] Farrell J., Kelly C., Rauch J., Kida K., García-Muñoz A., Monsefi N., Turriziani B., Doherty C., Mehta J.P., Matallanas D. (2014). HGF induces epithelial-to-mesenchymal transition by modulating the mammalian Hippo/MST2 and ISG15 pathways. J. Proteome Res..

[13] Bhowmick N.A., Ghiassi M., Bakin A., Aakre M., Lundquist C.A., Engel M.E., Arteaga C.L., Moses H.L. (2001). Transforming growth factor-beta1 mediates epithelial to mesenchymal transdifferentiation through a RhoA-dependent mechanism. Mol. Biol. Cell.

[14] Tsubakihara Y., Moustakas A. (2018). Epithelial-Mesenchymal Transition and Metastasis under the Control of Transforming Growth Factor β. Int. J. Mol. Sci..

[15] Manning C.B., Vallyathan V., Mossman B.T. (2002). Diseases caused by asbestos: Mechanism of injury and disease development. Int. Immunopharmacol..

[16] Tamminen J.A., Myllärniemi M., Hyytiäinen M., Keski-Oja J., Koli K. (2012). Asbestos exposure induces alveolar epithelial cell plasticity through MAPK/Erk signaling. J. Cell Biochem..

[17] Qi F., Okimoto G., Jube S., Napolitano A., Pass H.I., Laczko R., Demay R.M., Khan G., Tiirikainen M., Rinaudo C. (2013). Continuous exposure to chrysotile asbestos can cause transformation of human mesothelial cells via HMGB1 and TNF-α signaling. Am. J. Pathol..

[18] Pociask D.A., Sime P.J., Brody A.R. (2004). Asbestos-derived reactive oxygen species activate TGF-beta1. Lab. Investig..

[19] Maeda M., Chen Y., Hayashi H., Kumagai-Takei N., Matsuzaki H., Lee S., Nishimura Y., Otsuki T. (2014). Chronic exposure to asbestos enhances TGF-β1 production in the human adult T cell leukemia virus-immortalized T cell line MT-2. Int. J. Oncol..

[20] Gulino G.R., Polimeni M., Prato M., Gazzano E., Kopecka J., Colombatto S., Ghigo D., Aldieri E. (2016). Effects of Chrysotile Exposure in Human Bronchial Epithelial Cells: Insights into the Pathogenic Mechanisms of Asbestos-Related Diseases. Environ. Health Perspect..

[21] Xie F., Zhang Z., Van Dam H., Zhang L., Zhou F. (2014). Regulation of TGFβ Superfamily Signalling by SMAD Mono-Ubiquitination. Cell.

[22] Casarsa C., Bassani N., Ambrogi F., Zabucchi G., Boracchi P., Biganzoli E., Coradini D. (2011). Epithelial-to-mesenchymal transition, cell polarity and stemness-associated features in malignant pleural mesothelioma. Cancer Lett..

[23] Schramm A., Opitz I., Thies S., Seifert B., Moch H., Weder W., Soltermann A. (2010). Prognostic significance of epithelial-mesenchymal transition in malignant pleural mesothelioma. Eur. J. Cardiothorac. Surg..

[24] Kim M.C., Hwang S.H., Kim N.Y., Lee H.S., Ji S., Yang Y., Kim Y. (2018). Hypoxia promotes acquisition of aggressive phenotypes in human malignant mesothelioma. BMC Cancer.

[25] Katoh M., Katoh M. (2006). Cross-talk of WNT and FGF signaling pathways at GSK3beta to regulate beta-catenin and SNAIL signaling cascades. Cancer Biol. Ther..

[26] Zhou B.P., Deng J., Xia W., Xu J., Li Y.M., Gunduz M., Hung M.C. (2004). Dual regulation of Snail by GSK-3beta-mediated phosphorylation in control of epithelial-mesenchymal transition. Nat. Cell Biol..

[27] Peinado H., Olmeda D., Cano A. (2007). Snail, Zeb and bHLH factors in tumour progression: An alliance against the epithelial phenotype?. Nat. Rev. Cancer.

[28] Sullivan D.E., Ferris M., Pociask D., Brody A.R. (2008). The latent form of TGFbeta(1) is induced by TNFalpha through an ERK specific pathway and is activated by asbestos-derived reactive oxygen species in vitro and in vivo. J. Immunotoxicol..

[29] Miyazono K. (2009). Transforming growth factor-beta signaling in epithelial-mesenchymal transition and progression of cancer. Proc. Jpn. Acad. Ser. B Phys. Biol. Sci..

[30] Jope R.S., Yuskaitis C.J., Beurel E. (2007). Glycogen synthase kinase-3 (GSK3): Inflammation, diseases, and therapeutics. Neurochem. Res..

[31] Merikallio H., Pääkkö P., Salmenkivi K., Kinnula V., Harju T., Soini Y. (2013). Expression of snail, twist, and Zeb1 in malignant mesothelioma. APMIS.

[32] Saito A., Horie M., Nagase T. (2018). TGF-β Signaling in Lung Health and Disease. Int. J. Mol. Sci..

[33] Saito A., Horie M., Micke P., Nagase T. (2018). The Role of TGF-β Signaling in Lung Cancer Associated with Idiopathic Pulmonary Fibrosis. Int. J. Mol. Sci..

[34] Burmeister B., Schwerdtle T., Poser I., Hoffmann E., Hartwig A., Müller W.U., Rettenmeier A.W., Seemayer N.H., Dopp E. (2004). Effects of asbestos on initiation of DNA damage, induction of DNA-strand breaks, P53-expression and apoptosis in primary, SV40-transformed and malignant human mesothelial cells. Mutat. Res..

[35] Simeone P., Trerotola M., Franck J., Cardon T., Marchisio M., Fournier I., Salzet M., Maffia M., Vergara D. (2018). The multiverse nature of epithelial to mesenchymal transition. Seminars in Cancer Biology.

[36] Giribaldi G., Valente E., Khadjavi A., Polimeni M., Prato M. (2011). Macrophage inflammatory protein-1alpha mediates matrix metalloproteinase-9 enhancement in human adherent monocytes fed with malarial pigment. Asian Pac. J. Trop. Med..

